# Brain Network Changes in Fatigued Drivers: A Longitudinal Study in a Real-World Environment Based on the Effective Connectivity Analysis and Actigraphy Data

**DOI:** 10.3389/fnhum.2018.00418

**Published:** 2018-11-12

**Authors:** André Fonseca, Scott Kerick, Jung-Tai King, Chin-Teng Lin, Tzyy-Ping Jung

**Affiliations:** ^1^Center of Mathematics, Computation and Cognition, Federal University of ABC, São Paulo, Brazil; ^2^Swartz Center for Computational Neuroscience, University of California, San Diego, La Jolla, CA, United States; ^3^US Army Research Laboratory, Aberdeen, MD, United States; ^4^Brain Research Center, National Chiao Tung University, Hsinchu, Taiwan; ^5^Faculty of Engineering and Information Technology, Centre for Artificial Intelligence, University of Technology Sydney, Sydney, NSW, Australia

**Keywords:** drivers, fatigue, sleep, actigraphy, EEG, effective connectivity, Convergent Cross Mapping

## Abstract

The analysis of neurophysiological changes during driving can clarify the mechanisms of fatigue, considered an important cause of vehicle accidents. The fluctuations in alertness can be investigated as changes in the brain network connections, reflected in the direction and magnitude of the information transferred. Those changes are induced not only by the time on task but also by the quality of sleep. In an unprecedented 5-month longitudinal study, daily sampling actigraphy and EEG data were collected during a sustained-attention driving task within a near-real-world environment. Using a performance index associated with the subjects' reaction times and a predictive score related to the sleep quality, we identify fatigue levels in drivers and investigate the shifts in their effective connectivity in different frequency bands, through the analysis of the dynamical coupling between brain areas. Study results support the hypothesis that combining EEG, behavioral and actigraphy data can reveal new features of the decline in alertness. In addition, the use of directed measures such as the Convergent Cross Mapping can contribute to the development of fatigue countermeasure devices.

## 1. Introduction

Fatigue is a complex, dynamic, multidimensional construct involving subjective, behavioral, neural, and physiological processes that interact over varying timescales across a milieu of tasks and environmental contexts, making it difficult to operationally define and measure in a consistent or unitary way for scientific investigation. This study considers two different sources of fatigue operating on different timescales that interact in complex ways and vary both across individuals and within individuals over time. The first source of fatigue (or sleepiness) is related to circadian rhythms or sleep-wake cycles (sleep-related, e.g., acute or chronic sleep deprivation leading to sleep pressure) and the second source is related to the nature, complexity, and duration of the current task one is performing (task-related, e.g., task difficulty or demand, time-on-task which may lead to ones disinclination to continue performing a particular task). The importance of distinguishing between sleep- and task-related fatigue is that they reflect two conceptually distinct and separable sources of potential variations in performance and underlying brain mechanisms and require different mitigation strategies (May and Baldwin, 2009; Balkin and Wesensten, 2011). However, these underlying processes may interact in complex ways. Fatigue may lead to the decline of cognitive functioning and lapses in attention. It has cumulative and persistent effects in daytime performance (Belenky et al., 2003) and is considered a major factor in traffic accidents caused by human errors (Inoue and Komada, 2014).

Fatigue diminishes road safety, accounting for approximately 25% of car accidents (Brown, 1994) and 57% of commercial truck accidents (Bonnet and Arand, 1995). Young people around 20 years old are particularly vulnerable to fatigue-related accidents (Pack et al., 1995). Generally speaking, fatigue is also associated with increased stress and impaired cognitive performance at work (Härmä et al., 2006). The effects of fatigue can vary over various timescales depending on task and context, but are generally classified as acute (sudden onset, relieved by rest) or chronic (persistent, lasting from days to years) which vary from poor accomplishments to health and security problems (Spurgeon et al., 1997).

Understanding antecedents and consequences of fatigue and having a capability to predict fatigue-related performance decrements is a matter of public safety and wellness. When there is a risk of error or accident, the individual alertness and cognitive performance can be measured and the attention lapses can be putative. Specifically about drivers, biomathematical models have been developed to associate fatigue levels with working patterns. For instance, the circadian information, which is linked to task performance (Harrison et al., 2007), can be recorded from activity and rest periods and then processed by those models to estimate sleep quality and to infer sleep-related fatigue. From several biomathematical approaches we choose the *Sleep, Activity, Fatigue, and Task Effectiveness* (SAFTE) (Hursh et al., 2004), which records data of circadian rhythm, homeostatic drive, and sleep inertia, to characterize the sleep-awake history of drivers. The results of SAFTE has been validated as a neurobehavioral performance predictor in laboratorial and real-world environments (Dawson et al., 2011).

Another important resource to investigate and predict drivers' fatigue is the qualitative and quantitative EEG analysis, which has been used to unveil the relation between brain activity or brain network changes and the decline in alertness (Huang et al., 2015, 2016; Lin et al., 2016). The findings link behavioral performance with changes in EEG power spectrum and in the default mode network, suggesting that significant neural circuits must be activated to sustain performance and prevent attentional lapses. The investigation of those correlates is based on the brain connectivity theory and considering the alert-drowsiness transitions as an emergent effect of a complex system.

To analyze the underlying brain circuitry in the fatigue phenomenon, concepts of functional and effective connectivity can be applied. The first one refers to the statistical dependence in the neuronal activity and the second quantifies the influence that one brain area exerts over another (see Friston, 2011 and Goldenberg and Galvn, 2015 for definitions and techniques). Those concepts allow different interpretations and can be complimentary (Friston et al., 2013).

Functional connectivity can be undirected as in correlation and coherence measures, or directed as in *Granger Causality* (GC) (Granger, 1969) and transfer entropy (Schreiber, 2000). Multivariate extensions of GC such as directed transfer function (Kaminski and Blinowska, 1991) and partial directed coherence (Baccalá and Sameshima, 2001) allow time-varying and frequency-selective analysis (see Barnett and Seth, 2014 for theoretical basis and numerical simulation of several brain connectivity estimators based on GC). Effective connectivity measures consider the directed integration in neuronal macrocircuits as in the dynamic causal modeling (Friston et al., 2003). The methodology choice relies on the assumptions of the underlying mechanism.

In our analysis, we considered dynamic emergent effects from coupling variables and the effective connectivity approach was selected. The study was performed using the *Convergent Cross Mapping* (CCM) (Sugihara et al., 2012). CCM quantifies the directed interactions considering non-linear and linear components, stationary and non-stationaty features in bivariate or multivariate systems (McCracken and Weigel, 2014; Hirata et al., 2016; Jiang et al., 2016). CCM detects the causal relation strength and information exchanged between signals, assessing the synchronization features through the correspondence of the reconstructed phase-spaces, obtained from time-delay embedding coordinates. CCM has provided new insights into physiological states by considering the brain as a complex network system (McBride et al., 2015; Schiecke et al., 2017).

This work analyzed the brain network changes of drivers by the shifts in the effective connectivity expressed in the CCM oscillations. Moreover, this work investigated the modulation of the power spectra by those shifts. To assess possible CCM-power correlations, we first decomposed the EEG signals into different frequency bands prior to evaluating causal relations, providing information about effective connectivity changes for each neural rhythm. Using this procedure and the properties of dynamical coupling, it is plausible to assume that the CCM from the source signal to the target signal can modify dynamically phase and amplitude of the target observation. This principle is supported by fMRI studies such as Baechinger et al. (2017).

This methodology aims to detect changes in brain dynamics associated with the task-positive network of drivers to characterize alert and fatigue states during the simulated driving task. We combine EEG and non-EEG (subjective and behavioral data) recordings in the context of non-stationary data. For EEG signals, we choose to explore causal features in the reconstructed phase space considering the sources near the Frontal Midline and Parietal Midline brain areas. Our approach was based on the importance of dominating brain regions during driving to detect fluctuations in attention (Lin et al., 2016) and on the evidence of specific connectivity patterns in cortical regions related to behavioral microsleeps, a inherently non-stationary phenomena (Toppi et al., 2016).

The present study begins with a description of the subjects, the actigraphy data used to define levels of sleep-related fatigue and of the realistic sustained-attention experiment, detailing the EEG data, and *Reaction Time* (RT) acquisitions. The information of those sources was combined to test the hypothesis that driving performance impairment in fatigued drivers is associated with effective connectivity shifts. Second, we define a *Driver Performance* (DP) index, explained the phase-space reconstruction procedure (needed for the CCM evaluation) and presented a simulation study to test CCM efficiency in a brain connectivity model. Next, we describe the statically analysis of DP, CCM, and power values over the sleep-related fatigue levels. Finally, we show the results and conclude our work with a discussion of the different brain network patterns detected in the sleep-related normal and fatigued levels.

## 2. Materials and methods

### 2.1. Subjects

Seventeen healthy university students, 13 males and 4 females, with normal or corrected to normal eyesight, no neurological, or psychiatric disorders, aged 22.4± 1.5 years, all right-handed, from National Chiao Tung University (NCTU) in Taiwan participated in this study. The experimental protocol of the sustained-attention task was approved by the Institutional Review Board and written informed consent was obtained from each participant after a full explanation of the study.

### 2.2. Actigraphy data acquisition and fatigue level

As a part of a *Daily Sampling System* (DSS), the subjects used a wrist-worn device (Fatigue Science Readiband^TM^), which records circadian, homeostatic and sleep inertia processes on a minute basis. This device incorporates the information collected in the last 3 days and, applying the biomathematical model SAFTE, provides a putative performance level called *Effectiveness Score* (ES), which can be easily read from the device. Based on this score, we classified the subjects into three levels of sleep-related fatigue, *Normal* (NO) for ES greater than 90%, *Reduced Risk* (RR) for ES between 70 and 90%, and *High Risk* (HR) for ES smaller than 70%. The HR level of sleep-related fatigue represents a putative performance comparable to subjects with 0.08 blood alcohol level or awake for 21 h. For more information about the SAFTE model and ES use/validation see Hursh et al. (2004), Hursh et al. (2006), and Russell et al. (2006).

### 2.3. Experimental paradigm and sessions

**Figure 1:**
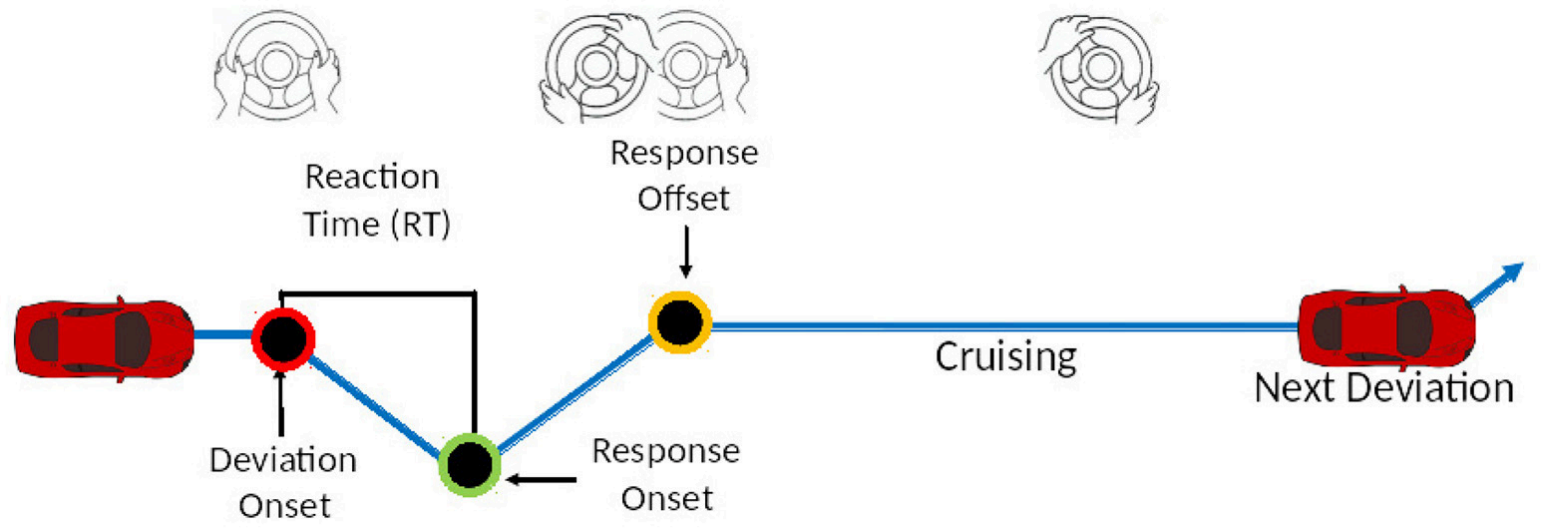
*Lane Keeping task* experiment. Subjects have to steer the wheel, when the realistic simulated vehicle is drifting away from the original cruising lane, to compensate the perturbation. There are no acceleration and brake controls; simulated cruising speed was kept constant at 45 mph.

In this sustained-attention experiment we adapted the *Lane Keeping Task* (LKT) as the driving paradigm (Huang et al., 2016), where subjects must maintain the cruising position on the central lane and compensate randomly induced vehicle deviations by turning the steering wheel (see Figure 1). The experiment was conducted at the Brain research Center at NCTU using a realistic driving simulator (Chuang et al., 2012). The ES of the subjects (reflecting the sleep quality of previous nights) were tracked and reported automatically. They were asked to come to the lab when a desirable score is detected, respecting a balance among the sleep-related fatigue levels NO, RR, and HR. Each LKT session lasted 30 min. Before it, they were instrumented with the EEG and asked to sit and stay quiet for 2 min. The experimental paradigm simulated a night-view cruising and the lane departures were equally distributed between left and right deviations. Perturbations were presented at intervals of approximately 1 every 7−12 s jittered to prevent anticipatory reactions of the drivers (resulting in approximately 180 events per session). If there is no response to the deviation, the simulated vehicle hits the curb and keeps its movement with no feedback to the subject.

During a longitudinal study spanning a 5-month period of daily sampling, 12 subjects were able to complete 3 sessions within each of the three levels of the ES. The rest of the participants completed at least 2 sessions within two classification levels. The subjects attended the sessions within 1−3 week intervals and the total number of completed EEG sessions was 141.

### 2.4. EEG data acquisition and preprocessing

A 64-channel EEG system (Neuroscan Inc.) was used to collect EEG data during the driving task, with channel locations measured by a 3D digitizer following the international 10-20 system. The sampling rate was 1,000 Hz and the impedance was kept below 5*KΩ* for all electrodes. The ocular and muscular artifacts were identified in epochs with an amplitude exceeding ± 70 μV (see Figure S1 in Supplementary Material for an example) and removed by visual inspection (Tatum et al., 2007; Tandle et al., 2016). The signals were band-pass filtered between 0.5 and 50 Hz and then downsampled to 500 Hz. For our analysis, we selected brain areas and respective channels described in the Table 1, based on Lainscek's study (Lainscsek et al., 2013). Our analyses focused on the EEG signals 1 s (or 500 points) before each lane-departure event. This choice aims to capture the tonic modulations of attention and engagement during a sustained performance in simulated driving tasks and it was based on the studies of Huang et al. (2007), Chuang et al. (2014), and Lin et al. (2016).

**Table 1:** Brain areas and the respective selected channels for the effective connectivity analysis.

**Areas**	**Channels**
Left anterior	F1 F3 F5
Right anterior	F2 F4 F6
Left motor	FC1 FC3 FC5 C1 C3 C5
Right motor	FC2 FC4 FC6 C2 C4 C6
Frontal midline	FCz Cz
Left parietal	CP3 CP5 TP7 P3 P5 P7
Right parietal	CP6 CP4 TP8 P4 P6 P8
Parietal midline	CPz Pz
Left occipital	PO3 PO7 O1
Right occipital	PO4 PO8 O2

*For each session, the measures applied in this work were derived from single trials, normalized to the baseline information and then averaged over channels*.

### 2.5. Hypotheses

We hypothesize that the lack of attention in drivers emerges from the interaction of neurobiological mechanisms associated with sleep- and task-related fatigue processes. More specifically, the performance decrements in fatigued drivers are accompanied by effective connectivity changes in several brain areas tied to different spectral behaviors associated with the real-world distractors, resulting in different patterns of the neural rhythms augmentation or suppression.

### 2.6. Reaction time and drive performance

Defined as the elapsed time between the lane departure onset and the response onset, the *Reaction Time* (RT) has been used by several studies to detect subjects' fluctuations of performance in the simulated driving tasks (Huang et al., 2016; Lin et al., 2016). Short RTs are expected from alert drivers who respond quickly to cruising perturbations whereas drowsy drivers tend to react slower and produce longer RTs. To alleviate inter- and intra-subject variability, we define a *Normalized Reaction Time* (NRT) dividing the RTs by the average of the 10% shortest values within each session (sorted in ascending order). For our analyses, we consider a RT lower bound 1 s and upper bound 4 s to analyze transitions from alert to drowsy states. Subjects with NRT out of this interval are considered in very high or very low vigilance states. In the literature, significant changes in power spectra and in directed measures were empirically observed between 2 and 3 s (Chuang et al., 2012; Huang et al., 2015, 2016; Lin et al., 2016). We used a logistic transformation to rescale the NRT to those limits, defining a *Driving Performance* (DP) index (Huang et al., 2015):

$$\begin{array}{l} DP\left(NRT\right)=\frac{2+2e^{-0.5}}{\left(1+e^{-0.5NRT}\right)\left(1-e^{-0.5}\right)}-\frac{1+e^{-0.5}}{1-e^{-0.5}}. \end{array}$$

Notice that *DP*(1) = 1, DP tends to approximately 4.08 as NRT tends to infinity and it exhibits a close linear relation for NRT between 1 and 4. After the transformation, we set *DP* = 1 for *DP* < 1 and *DP* = 4 for *DP*>4. Therefore, DP maps the unbounded NRT to the interval [1, 4].

### 2.7. Phase-space reconstruction

Given an EEG signal, *X* = {*x*_1_, …, *x*_*n*_}, the spatial and time-delayed embedding coordinates are defined as $X_{vec}=\left\{\vec{x_{i}}=\left(x_{i},x_{i+\tau ,},\ldots ,x_{i+\left(m-1\right)\tau }\right);i=1,\ldots ,N\right\}$ where *N* = *n*−(*m*−1)τ. The embedding parameters *m* and τ can be determined independently using the non-parametric Kozachenko-Leonenko estimator (Kozachenko and Leonenko, 1987), as done by *Gautama, Mandic and Hulle* (GMH) (Gautama et al., 2003). This procedure avoids oversampled trajectories and autocorrelated data effects (Kennel and Abarbanel, 2002). Using the GMH approach for the EEG signals from all subjects and sessions (more details and applications in Baggio and Fonseca, 2011; Fonseca et al., 2015), we obtained *m* = 4 and τ = 1, respectively the maximum embedding dimension and minimum time lag found (see section 3.4 in Supplementary Material for the reconstruction Matlab script).

### 2.8. CCM

Given two EEG signals *X*, *Y* with length *n*, we calculate the phase space reconstruction coordinates *X*_*vec*_ with embedding parameters *m* and τ . For *i* = 1, …, *N* where *N* = *n*−(*m*−1)τ, we consider each vector $\vec{x_{i}}$ (representing the system dynamical evolution) and obtain:

1 - the distances from $\vec{x_{i}}$ to all other states in *X*_*vec*_: $D_{i}=\left\{d\left(\vec{x_{i}},\vec{x_{j}}\right)\;,i\neq j\right\}$, where *d* represents the euclidean distance between vectors.

2 - the distance-related weights: $u_{i}=e^{\frac{-d\left(\vec{x_{i}},\vec{x_{j}}\right)}{min}}$, where *min* is the minimum distance found in *D*_*i*_ calculations.

3 - the normalized weights: $w_{i}=\frac{u_{i}}{\overset{N-1}{\underset{j=1}{\sum }}u_{j}}$.

4 - the scalar *y*-value estimated by *X*_*vec*_: $\hat{y_{i}}=\overset{N-1}{\underset{j=1}{\sum }}w_{j}y_{j}$.

We define the CCM from the source signal *X* to the target signal *Y*, as the correlation between Ŷ = {ŷ_1_, …, ŷ_*N*_} and *Y* = {*y*_*n*−*N*+1_, …, *y*_*n*_} where *N* = *n*−(*m*−1)τ.

Notice that steps 1 to 3 are about *X* information and, in step 4, we use the temporal correspondence between *X*_*vec*_ and *Y*_*vec*_ to predict *Y* information, where the weights defined in step 3 are the highest for the closest neighbors. By definition, CCM is asymmetric and lies in the interval [−1, 1] (see section 3.5 in Supplementary Material for the CCM main Matlab script).

**Figure 2:**
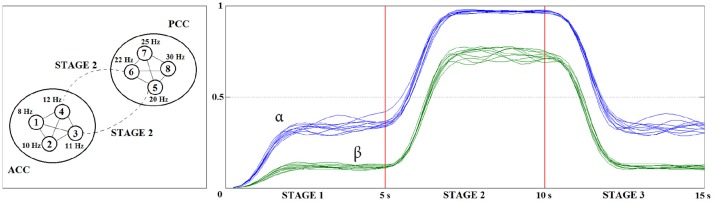
Illustration of the simulated eight dynamically coupled sources **(Left)** from the brain connectivity model performed in three stages of 5 s each. On stages 1 and 3 the alpha cluster in ACC and beta cluster in PCC are only intra-coupled. On stage 2, the clusters are intra- and inter-coupled. The source activations were projected to the scalp using a BEM forward head model. The 64-channel EEG signals were simulated for 10 different levels of noise and then decomposed into the alpha and beta bands. Averaged CCM values from anterior to posterior channels **(Right)** were consistent with the changes in the inter-cluster coupling during the stages.

To test the efficiency of CCM, following the ideas reported in Ball et al. (2016), we designed a brain connectivity model with eight coupled damped oscillators sources (see Figure 2, left panel) defined by *Autoregressive Processes* (AR) of order 5. Sources 1 to 4 are coupled and located in the *Anterior Cingulate Cortex* (ACC) with respective rhythms 8, 10, 11, and 12 Hz, defining an alpha cluster. Sources 5 to 8 are coupled and lie in the *Posterior Cingulate Cortex* (PCC) with respective frequencies 20, 22, 25, and 30 Hz, configuring a beta cluster. The simulation was performed in three stages of 5 s each. ACC and PCC clusters are disconnected in stages 1 and 3 and coupled during stage 2. Intra- and inter-cluster couplings were defined by Gaussian mixture AR models.

Aiming the analysis of the changes at the causal relationship between the ACC and PCC clusters in the channel level, we used a *Boundary Element Method* (BEM) from the SIFT toolbox (Mullen, 2012) to generate 64-channel EEG signals. This realistic forward head model projects the source activations to the scalp using the “colin27” brain atlas as the reference (Holmes et al., 1998). Varying the white-noise variances in the AR processes from 0.1 to 1 s (step 0.1 s), we simulated ten 64-channel EEG signals with the sampling frequency of 200 Hz (see sections 3.1 to 3.3 in Supplementary Material for the SIFT settings).

We decomposed the signals into the alpha and beta bands and calculated CCM in windows of 0.25 s (20 points per stage) from the channels in the Left and Right Anterior areas to the ones in the Left and Right Posterior areas (see Table 1). The averaged CCM over channels (see Figure S2 in Supplementary Material for the flowchart of simulation study). is plotted for each noise level in Figure 1, right panel. The observed changes in the causal outflow from anterior to posterior channels are consistent with the brain connectivity model defined at the source level. CCM is robust to noise and insensitive to linear mixtures.

### 2.9. Statistical analysis

Considering 141 sessions and an average number of 143 events per session (total of 20, 182 events), we checked the statistical significance of CCM from source to target areas selected in this work. We performed a bootstrapping approach using surrogate data with the same power spectrum of the original signals (Baggio and Fonseca, 2011). A Wilcoxon rank sum test was used with 1% significance level to verify the null hypothesis that the original data, epochs of 1 s before the events for each subject and session, and its surrogates have the same distribution of the CCM values. The null hypothesis was rejected for all sessions indicating that the causal relations are a genuine non-linear feature of the data.

**Figure 3:**
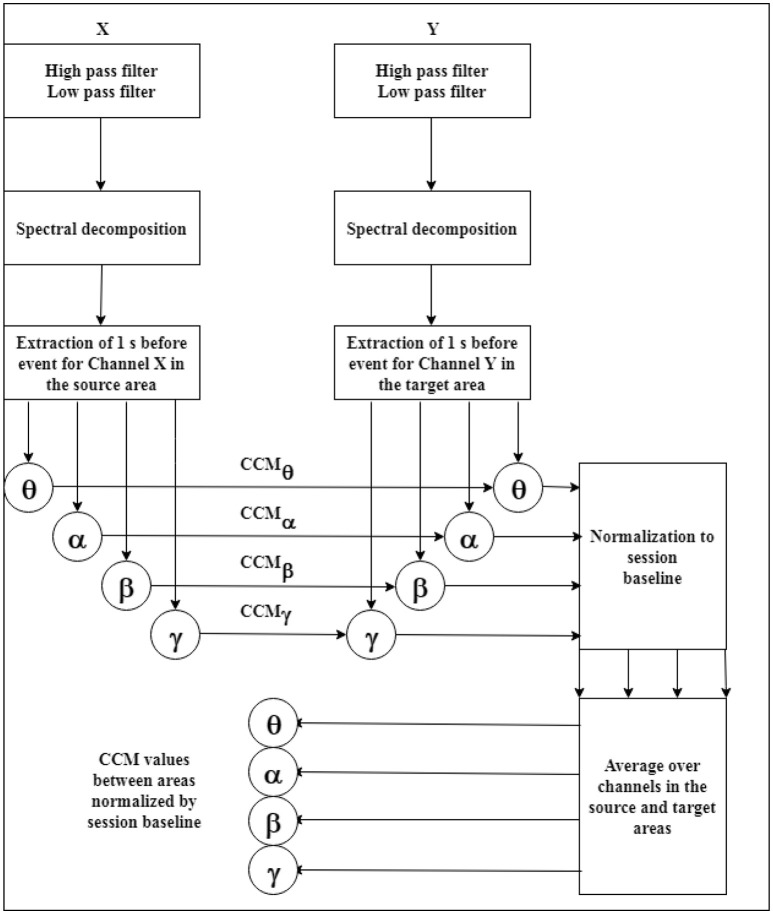
Event signal processing pipeline for the CCM values from source channels *X* to target channels *Y*. The power analysis for the target channels was conducted using the same steps.

For each session and event, the signals were decomposed into the bands θ:[4.5 , 7.5] Hz; α:[7.5 , 12.5] Hz; β:[12.5 , 20] Hz and γ:[25 , 40] Hz, using a FFT procedure. For each band, CCM from source to target channels were calculated in the sessions. Considering in each session the baseline set as the CCM values corresponding to the 10% shortest DPs (in ascending order), CCM were normalized by subtracting the median and dividing by the quartile dispersion of the baseline set. Then, the normalized CCM values were averaged over channel pairs belonging to source and target areas (see Table 1 for the channel sets definition), defining a baseline relative causal relation between areas. See Figure 3 for the event signal processing pipeline. The same pipeline was applied to the spectral analysis calculations considering only the target channels *Y*. The spectral analysis was performed using the FFT procedure in Matlab (2012b). The results presented in this work will be always relative to the baseline set within sessions and averaged over channels.

The normalized CCM and spectral values from all subjects and sessions were aggregated, sorted by DP, and then separated in the three levels of sleep-related fatigue NO, RR, and HR with respective sample sizes of 6811, 7136, and 6235.

The significant statistical difference for the normalized CCM values between categories was analyzed by two criteria: the distribution difference was validated by the Wilcoxon rank sum test with 1% significance level, and the slope difference was checked by the *F*-test with 1% significance level as well.

CCM-DP, power-DP, and CCM-power statistical relations were investigated by the Pearson's correlation (see Figure S3 in Supplementary Material for a flowchart of the overall process).

## 3. Results

### 3.1. NRT and DP distributions

**Figure 4:**
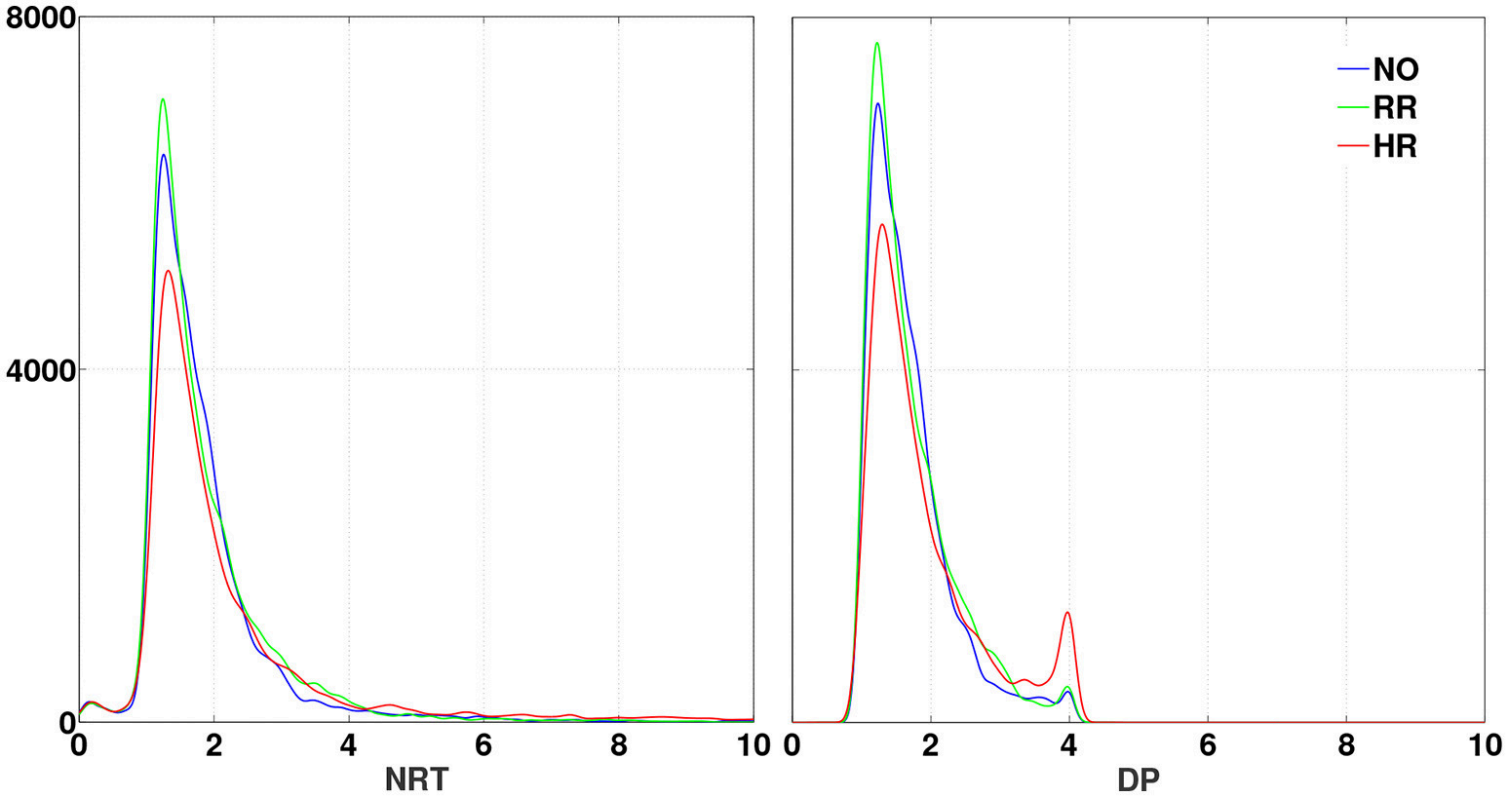
PDFs of NRT **(Left)** and DP **(Right)** for the sleep-related fatigue levels NO (blue), RR (green), and HR (red). All distributions are super-Gaussian like. The DP distributions exhibit a second peak, which is the highest for the HR level of sleep-related fatigue. The density was estimated at every 100 points.

The RTs were extracted from lane departure events for the 17 subjects and under three different sleep-related fatigue levels (defined by the quality of sleep). For each session, the NRTs were derived and then the DP indexes were obtained. Table 2 shows the descriptive statistics across NO, RR, and HR conditions defined by the ES. The NRT and DP distributions are skewed to the right due to slow reactions of fatigued drivers and the experimental paradigm (no feedback for hitting the curb). Their distributions are super-Gaussians (Lee et al., 1999) with one and two peaks, respectively, as shown in Figure 4. The logistic transformation in the DP calculation was able to decrease the normalized reaction time variance and keep the quartile dispersion in the same order of magnitude than NRTs, i.e., a non-linear transformation with close to linear effects. The conversion from NRT to DP is a useful procedure for correcting experimental distortions and rescaling an unbounded measure to a more practical behavioral performance index.

**Table 2:** Descriptive Statistics of NRT and DP across the sleep-related fatigue levels NO, RR, and HR.

	**Normal**	**Reduced risk**	**High risk**
**Number of events**	6811	7136	6235
**NRT**			
Mean	1.9172	2.0589	2.8199
Standard deviation	1.6897	3.2471	9.2638
Quartile dispersion	0.2331	0.2581	0.2935
**DP**			
Mean	1.6873	1.7046	1.8858
Standard deviation	0.5986	0.6274	0.7987
Quartile dispersion	0.22097	0.2315	0.2579

*The transformation NRT to DP provides distributions with lower variability, but with similar quartiles dispersion structure*.

Also shown in Figure 4, the NRT-DP transformation keeps the ascending order among the sleep-related fatigue levels, for the NRTs- and DPs- distribution means and the peaks (lower values for HR, middle for NO and higher for RR). In the DP domain, it is clearly seen a higher probability of 4 (drowsy state) in the HR level of sleep-related fatigue, not noticed in the NRT domain. The DPs fit the interval [1, 4] (by definition) and reveal new features in the changes of alertness levels.

### 3.2. CCM oscillations indexed by DP

**Figure 5:**
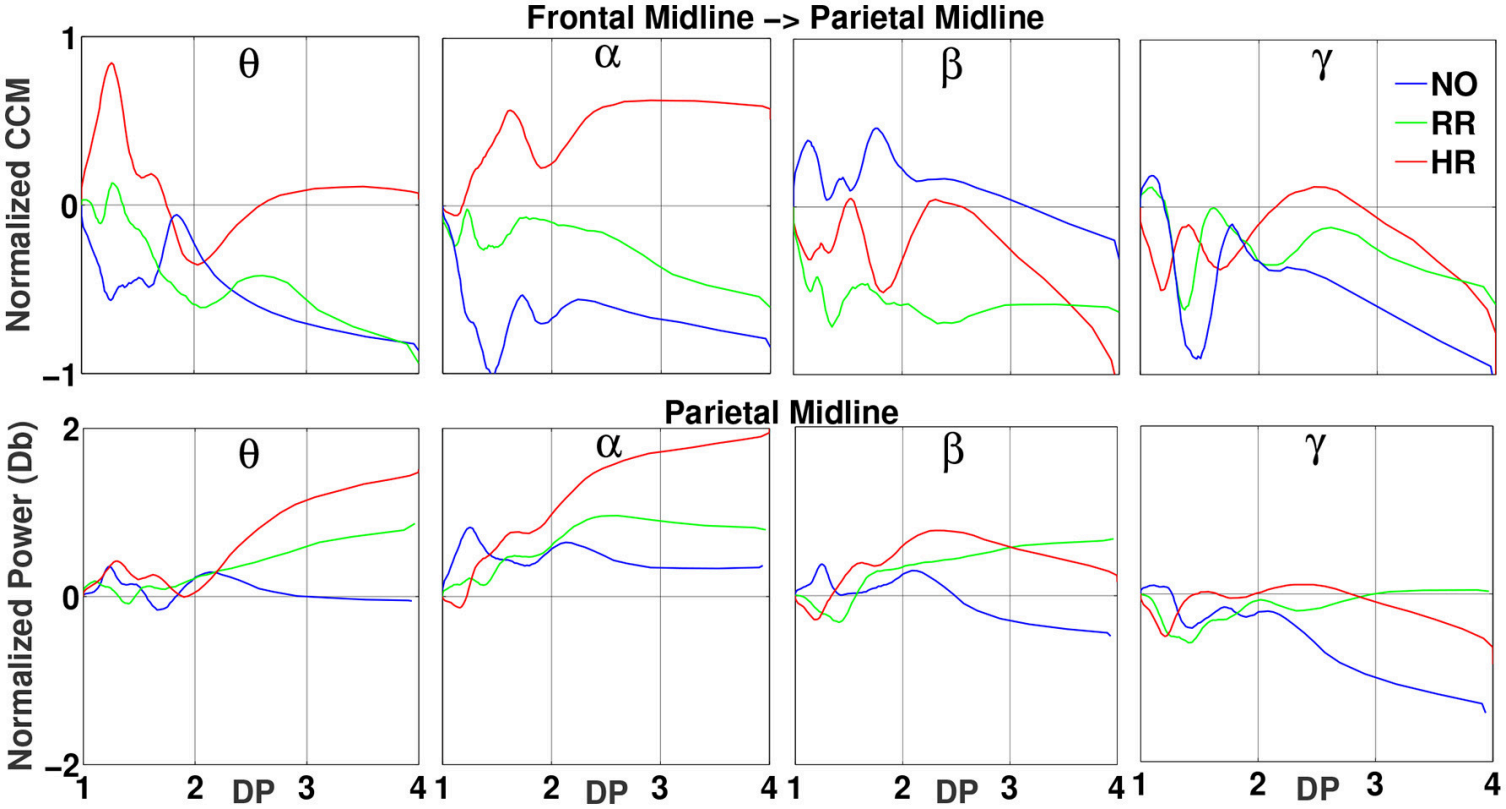
On the top are the normalized CCM values from the source area Frontal Midline to the target area Parietal Midline, sorted by DP . The bottom panels are the normalized power for the target area, sorted by the same index. The EEGs considered were 1 s (or 500 data points) before lane-departure events and decomposed into four brain rhythms. The subjects were classified by their ES into three sleep-related fatigue levels: NO (blue), RR (green), and HR (red). Possible correlations between normalized CCM and spectral values in the same band were investigated in this work. The measures were averaged across each 100 events, with standard deviation less than 10% of the mean value, and a moving-average filter with a window size of 0.5 s and a step size of 0.1 s was applied.

We first analyzed the relation between the normalized CCM and DP values. For different target areas and bands, the CCM values exhibit a strong oscillatory behavior in DP between 1 and 2. For DP between 2 and 4, a nearly monotonic behavior was noticed and the Pearson's correlation was evaluated. Considering the two categories of sleep-related fatigue NO and HR, for more than 90% of the 144 cases (2 source areas × 9 target areas × 4 frequency bands × 2 categories), the causal relations exhibited a strong positive or negative correlation (absolute value greater than 0.7) with the performance index DP. The strong correlation for the RR level of fatigue was not observed in this case. In the Figure 5, the top panel shows the normalized CCM values from the source, Frontal Midline, to the target, Parietal Midline areas, sorted by DPs, for the four frequency bands and three levels of sleep-related fatigue NO (blue), RR (green), and HR (red). In short DP's, between 1 and 2, related to alert states, it's possible to observe a mirror pattern between NO and HR levels. In the longer DP's, related to drowsy states, we observe different trends in the nearly monotonic behavior between the same two levels.

Table 3 shows the significant changes in the normalized CCM values of sleep-related fatigue levels NO vs. HR, for DPs between 2 and 4, where a nearly monotonic behavior was observed. The source of the dynamical coupling was the Frontal Midline area and the targets were the other selected areas represented in different rows. For several targets and bands, the HR- and NO-normalized CCM values have different distributions and slopes. Gray background cells in Table 3 indicate simultaneous significantly statistical differences in distributions and slopes (considering the significance level of 1%) between the two levels of sleep-related fatigue, i.e., it points out the targets and bands where the causal relations have different trends (positive and negative slopes) with different probability of occurrences.

**Table 3:** Statistical analysis of normalized CCM values in different bands (columns), considering the Frontal Midline area as the source and the other areas (rows) as targets.

		**Source: Frontal Midline**		
**Targets**	θ	α	β	γ
	**NO** **HR** *p*	**NO** **HR** *p*	**NO** **HR** *p*	**NO** **HR** *p*
Left anterior	−0.4455, 0.5050 (< 10^−4^)	0.1723, 0.6949 (< 10^−4^)	−0.6168, 0.8107 (< 10^−4^)	−1.7217, 0.5473 (< 10^−4^)
	−0.0363, 0.7376 (< 10^−4^)	−0.1934, 0.3099 (< 10^−4^)	−0.1934, 0.3099 (< 10^−4^)	−1.863, 0.2306 (< 10^−4^)
Right anterior	−0.6473, −0.2415 (< 10^−4^)	0.2379, 0.3733, (0.0040)	−0.0364, 0.5440, (< 10^−4^)	−0.6130, 0.8925, (< 10^−4^)
	−0.1636, −0.0306 (0.0139)	−0.1444, −0.01206 (0.0190)	−0.1172, 0.2219, (< 10^−4^)	−0.0956, 0.71532 (< 10^−4^)
Left motor	−0.3352, −0.2282 (0.3771)	0.5908, 0.7794 (0.4944)	−0.5266, 0.2940 (< 10^−4^)	−0.8709, 0.2390, (< 10^−4^)
	−0.0032, 0.4066 (< 10^−4^)	−0.1753, 0.6630 (< 10^−4^)	−0.2516, −0.2072 (0.2020)	−0.5131, −0.2163 (< 10^−4^)
Right motor	−0.7234, 0.2485 (< 10^−4^)	−0.1045, 0.37740 (0.0015)	−0.1504, −0.0984 (0.0343)	−1.2960, 0.7357 (< 10^−4^)
	−0.1144, 0.9210 (< 10^−4^)	0.3059, 0.6328 (< 10^−4^)	−0.1367, −0.0882 (0.0717)	−0.6241, 0.2950 (< 10^−4^)
Left parietal	−0.4649, −0.5567 (0.582)	0.4727, 0.6611 (0.4533)	−0.6212, 0.1600 (< 10^−4^)	−0.4561, 0.2505 (< 10^−4^)
	−0.3071, 0.1694 (< 10^−4^)	−0.2654, 0.7004 (< 10^−4^)	−0.2999, −0.0092 (< 10^−4^)	−0.1184, −0.1096 (0.8348)
Parietal midline	−0.5704, −0.0533 (< 10^−4^)	−0.6464, 0.5359 (< 10^−4^)	0.0687, −0.3857 (0.0001)	−0.5312, −0.1902 (0.0026)
	−0.2611, 0.1842 (< 10^−4^)	−0.1209, 0.0839 (< 10^−4^)	−0.2252, −0.6031 (0.0002)	−0.3546, −0.4453 (0.2083)
Right parietal	−0.7648, −0.4996 (0.0029)	−0.8135, 0.4037 (< 10^−4^)	−0.06380, −0.4208 (< 10^−4^)	−0.9670, 1.1025 (< 10^−4^)
	0.1465, 0.3360 (< 10^−4^)	0.2103, 0.2148 (0.9183)	0.2122, −0.2697 (< 10^−4^)	−0.4766, 0.3390 (< 10^−4^)

*For the sleep-related fatigue levels NO and HR, the distributions and slopes were analyzed for DP in the interval [2, 4]. In each cell, on the top, are the CCM means, respectively of NO and HR categories, and the p-value for the Wilcoxon rank test inside brackets, with the null hypothesis that the two levels of sleep-related fatigue have CCM values with the same distributions. On the bottom, are the slopes respectively of NO and HR values and the p-value for the F-test inside brackets, with the null hypothesis that those two levels have CCM values with identical slopes in their linear regressions. Simultaneous significant probabilities shift and trend changes between NO and HR levels are indicated by the gray background. See Figure 5 (top) to visualize plots of CCM from Frontal Midline to Parietal Midline areas*.

We also investigated the effective connectivity from the source, Parietal Midline, to the other selected targets. Although CCM is not symmetric by definition, the causal relations from the Parietal Midline to Frontal Midline are similar to its opposite direction values, indicating a bi-directional causation between those two areas. Table 4 shows the statistical analyses of the normalized CCM values from the Parietal Midline area between levels NO and HR, to different targets at different frequency bands, as shown in Table 3.

**Table 4:** Statistical analysis for normalized CCM values between NO and HR levels of sleep-related fatigue in different bands (columns), considering the Parietal Midline area as the source.

		**Source: Parietal Midline**		
**Targets**	θ	α	β	γ
	**NO** **HR** *p*	**NO** **HR** *p*	**NO** **HR** *p*	**NO** **HR** *p*
Left motor	−0.3324, −0.6485 (< 10^−4^)	−0.1870, 0.8239 (< 10^−4^)	−0.0292, −0.5834 (0.0003)	−0.3783, 0.16174, (< 10^−4^)
	−0.1417, 0.1447 (< 10^−4^)	−0.5294, 0.0463 (< 10^−4^)	−0.2978, −0.7328 (0.0003)	−0.5198, −0.2839 (0.0038)
Right motor	−0.2939, −0.5015, (0.0002)	−0.5207, 0.6376, (< 10^−4^)	−0.2758, −0.3164, (0.6054)	−0.8973, 0.6191 (< 10^−4^)
	0.2246, 0.1532 (0.0126)	−0.2958, −0.0573 (< 10^−4^)	−0.1883, −0.1997 (0.8385)	−0.4069, 0.2053 (< 10^−4^)
Left parietal	−0.2060, −0.6120 (0.0002)	0.0133, 0.8920 (< 10^−4^)	0.1918, 0.9166 (< 10^−4^)	−0.8330, −0.0814 (< 10^−4^)
	−0.0516, 0.4103 (< 10^−4^)	−0.1737, 0.1325 (0.0072)	−0.1124, 0.4233 (< 10^−4^)	−1.1663, −0.1527 (< 10^−4^)
Right parietal	−0.9579, −0.2734 (< 10^−4^)	−0.3390, −0.0357 (< 10^−4^)	−0.3526, 0.0388 (0.0023)	−0.4815, 0.9359 (< 10^−4^)
	0.4247, 0.1958 (< 10^−4^)	−0.1048, −0.1430 (0.1833)	0.3601, −0.4709 (< 10^−4^)	−0.1518, 0.2458 (< 10^−4^)
Left occipital	0.9939, −0.7421 (< 10^−4^)	−0.8257, 0.5110 (< 10^−4^)	−0.3524, 0.3047 (< 10^−4^)	−0.4807, −0.4323 (0.4944)
	0.9123, −0.1092 (< 10^−4^)	−0.4302, 0.1268 (< 10^−4^)	−0.2363, −0.2011 (0.3262)	−0.6942, −0.2405 (< 10^−4^)
Right occipital	−1.0210, −0.0349 (< 10^−4^)	−0.7530, −0.4193 (< 10^−4^)	−0.9199, −1.4260 (0.6054)	−1.2437, −0.1614 (< 10^−4^)
	0.0993, 0.0103 (< 10^−4^)	−0.1763, −0.1638 (0.7843)	−0.2920, −2.0188 (< 10^−4^)	−0.9254, −0.4901 (< 10^−4^)

*The parameters and p-values are the same defined in Table 3. As done before, simultaneous significant probabilities shift and trend changes are indicated by the gray background*.

### 3.3. Spectral power indexed by DP

The relation between normalized EEG power and DP was studied for all ten areas defined in Table 1, for the bands δ, α, β, γ, and for the two categories of sleep-related fatigue NO and HR. In Figure 5, for instance, the bottom panel shows the fluctuations of the normalized power in different bands, for the Parietal Midline area.

As for the normalized CCM values, the normalized power in the targets exhibits an oscillatory behavior for DP less than 2. For the DP higher than 2, we observed a nearly linear behavior. In this domain, between 2 and 4, the Pearson's correlation between power and DP were calculated and the results exhibited a strong positive or negative correlation (absolute value greater than 0.75) between spectral activity and the performance index for more than 90% of the 80 cases analyzed.

### 3.4. CCM-power correlation

After exploring the CCM-DP and power-DP relations, the next question is how CCM and spectral power interacted considering the same target area. The procedure of evaluating the causal relation to a specific target in different frequency bands allows a natural connection with spectral power in the same target. Considering the source of CCM as the Frontal Midline and Parietal Midline areas, we restricted our study to the cases where the distributions and slopes were significantly different between the levels of sleep-related fatigue NO and HR, marked as gray in Table 3. Table 5 lists the correlations between normalized CCM values (considering both sources) and normalized spectral power sorted by DPs.

**Table 5:** Pearson's correlations for the normalized CCM-spectral values considering the same target areas in different frequency bands for the sleep-related fatigue levels NO and HR.

**Targets**		**NO**	**HR**
**SOURCE: FRONTAL MIDLINE**			
Left anterior	θ:	0.5622	0.9867
	α:	0.9974	0.0684
	β:	0.9773	−0.9129
	γ:	0.9617	−0.9387
Right anterior	β:	0.7320	−0.8859
	γ:	0.0815	−0.9877
Right motor	θ:	0.8766	0.9491
	γ:	0.9920	−0.9716
Parietal midline	θ:	0.7685	0.9666
	α:	0.5330	0.9933
Right parietal	β:	−0.9527	0.9676
	γ:	0.9938	−0.7273
**SOURCE: PARIETAL MIDLINE**			
Left motor	θ:	0.9319	0.9399
	α:	0.9861	0.9038
Right motor	γ:	0.9788	−0.8707
Left parietal	θ:	0.6080	0.9916
	α:	0.9180	0.1632
	β:	0.6131	−0.9460
Right parietal	β:	−0.9363	0.9834
	γ:	0.9389	−0.9494
Left occipital	θ:	−0.8264	−0.6177
	α:	0.9674	0.6303

*On the left, the source of CCM is the Frontal Midline Area. On the right, the source is the Parietal Midline Area. The first column of each Table specifies the target areas. The choice was based on the simultaneous significant differences in distributions and slopes between those two levels (marked as gray in Tables 3, 4). Both measures were sorted by DPs within the interval [2, 4]. See Figure 5 for the plots of the case from the Frontal Midline source to the Parietal Midline target*.

## 4. Discussion

This study observed a group of young university students in their natural environment during a 20-week semester. We believe our subjects are a representative sample of healthy young adults in real-world environments, with expected high levels of stress and irregular sleep (Lund et al., 2010). With the sustained-attention experiment, we aim to understand the connections between those subjective parameters and the performance decrements in sleep-related fatigue, characterizing its variability and instability (Chua et al., 2014). To achieve this goal, the starting point was the signal-reconstruction process. The embedding coordinates revealed different recurrence structures linked to the three levels of sleep-related fatigue defined by the ES. We consider that this representation was sensible to the different quality and quantity of sleep across subjects, quantifying behavioral and physiologic information from the different fatigue states determined by the Readiband using the SAFTE model.

The choice of the performance index to sort the normalized CCM and power spectrum values was crucial. The NRTs exhibit high variance and positive-skew distributions, an expected outcome since fatigued drivers can exhibit low performance, failures (Huang et al., 2009; Liu et al., 2010), and even fall asleep. As the subjects have no feedback from the driving simulator when the vehicle hits the curb and maintains a continuous cruising, the NRTs can deviate significantly from the baseline. The transformation from NRTs to the DPs, considering the interval [1, 4] to analyze the EEG correlates of alertness-drowsiness transitions, alleviates this issue. As a consequence of its nearly linear behavior we obtain lower standard deviations, but with no robust changes in the data structure observed in the quartile dispersion (see Table 2). The DPs-distributions have two peaks, the second peak can be attributed to the drowsiness state. For the HR level of sleep-related fatigue, the second peak is the highest, which is consistent with the putative fatigue level derived from the actigraphy data (ES).

Both normalized CCM and spectral values were strongly correlated (positively or negatively) with DPs between 2 and 4 in the levels NO and HR of sleep-related fatigue, as illustrated in Figure 5. For the shorter DPs (lying in the interval [1, 2]) when subjects were in the alert state under the sleep levels NO and HR, different oscillations in several sources and targets were observed, a mirror behavior, indicating opposite shifts in the effective connectivity. As for longer DPs (lying in the interval [2, 4]), where subjects were drowsy, different trends and distributions for the CCM values were found between the NO and HR sleep levels, revealing again different shifts of the effective connectivity. Those results demonstrate that DP is an efficient index to understand alertness-drowsiness transitions (Huang et al., 2015).

The information transferred from the source areas Frontal Midline and Parietal Midline to their neighboring areas during the 1 s pre-stimulus period have different rates between subjects in the NO and HR levels of sleep-related fatigue. This difference can be attributed to specific patterns in the effective connectivity related to behavioral microsleeps, reported in Toppi et al. (2016). In both Frontal Midline and Parietal Midline sources of connectivity, for almost all analyzed targets (with the exception from the Parietal Midline area to the Right Occipital area) the normalized CCM values, in some frequency, have significantly different distributions, a negative slope in the NO condition and a positive slope for the HR of fatigue, indicating the ES classification (related to sleep quality) can distinguish new features in the fatigued drivers (with DPs between 2 and 4). In the HR fatigue level, for the bands indicated in the gray background cells in Tables 3, 4, the normalized CCM values increase with the increments in DP (with 3 exceptions), suggesting enhanced coupling among the studied areas in the fatigued drivers with low sleep quality.

The correlations between the normalized CCM and spectral values are detailed in Table 5 and represent a novel application to analyze the shifts in the effective connectivity in brain areas during the sustained-attention tasks, allowing us to explore its correlates with the subject fatigue level. We considered only the couplings and bands where study results showed significant differences in distributions and slopes of the causal relations between the sleep-related fatigue categories. Strong positive and negative values were derived.

**Figure 6:**
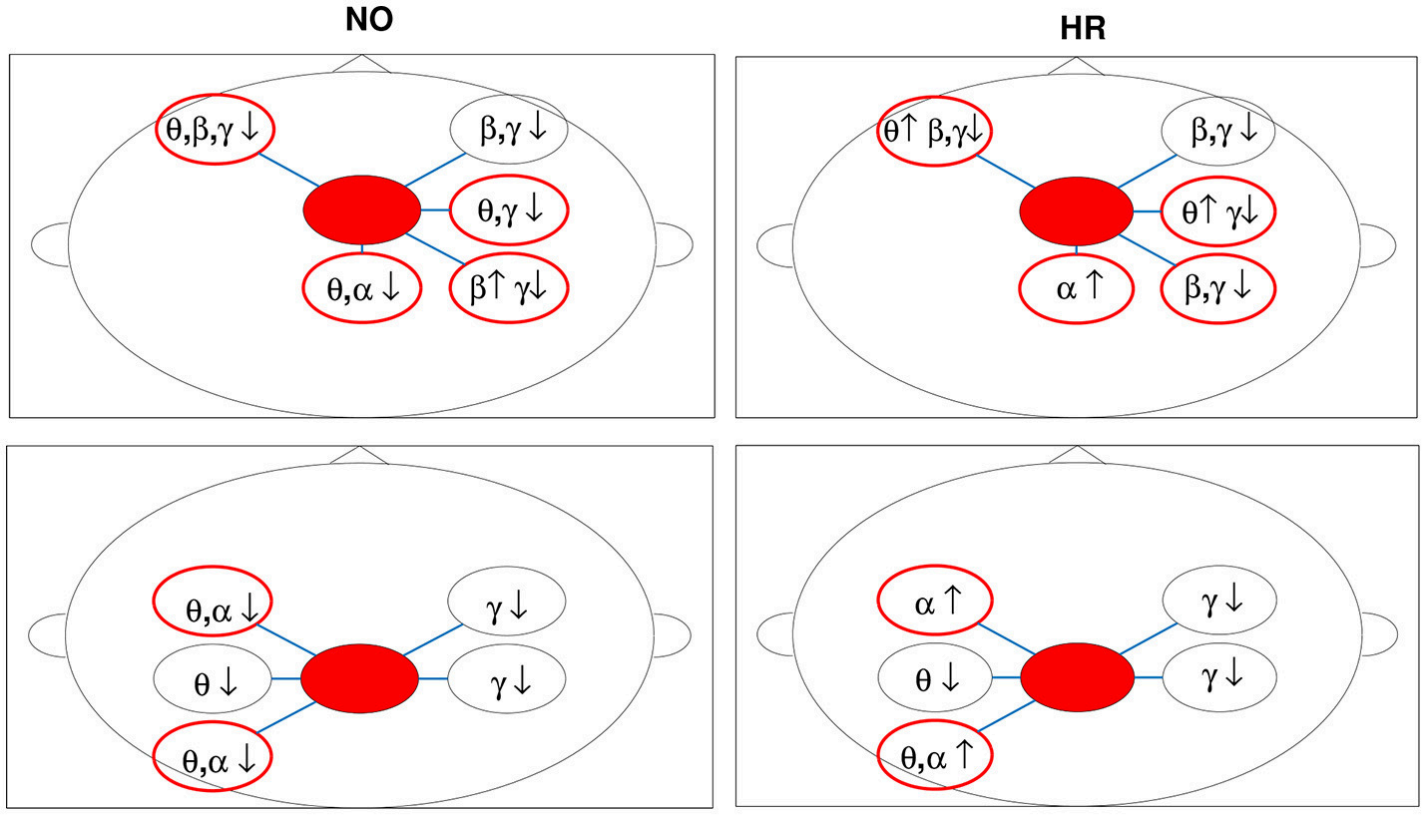
Brain network changes for fatigued drivers (grand averages for DPs between 2 and 4) in the sleep-related fatigue levels NO (good quality sleep) and HR (poor quality sleep). The Frontal Midline and Parietal Midline areas were considered sources (red filled circles) of the effective connectivity and the CCM-power correlations were analyzed. Augmentation and suppression in the neural rhythms are indicated respectively by up and down arrows. The red circles indicate target areas with different spectral activity (augmentation-suppression) between levels.

The normalized CCM values sorted by DP with an increasing magnitude indicates tonic changes of brain dynamics associated with a decline in alertness (DP variation from 2 to 4 is associated with sub-optimal and poor performances; Huang et al., 2015). We focused our attention on those cases, where the CCM values either increased with DP (a positive slope) or decreased with DP (a negative slope). The effective connectivity measure applied in this work is based on the dynamical coupling of brain areas and can modulate the power spectra as reported in Soldatenko and Chichkine (2014) and Lacot et al. (2016), where new power peaks and the enhancement of the original harmonics are associated with the increasing of coupling strength. In brain networks, this modulation was noticed in the BOLD signal analysis, where fMRI-based connectivity and frequency-specific EEG power are related (Conner et al., 2011; Scheeringa et al., 2012). So, it is reasonable to claim that strong CCM-power correlations represent augmentation or suppression for a specific oscillatory activity in the target areas. Taking this into consideration, we combined the information from Tables 3–5 and illustrated the brain network changes for the NO and HR levels of sleep-related fatigue in Figure 6. In the figure, the sources are indicated by the filled red circles and the augmentation or suppression are represented, respectively, by up and down arrows. The targets with significant differences between levels (augmentation to suppression or vice-versa) are indicated by red circles.

The γ band relates to the higher-order cognitive activities for internal modeling of motor control to form a representation shaping internal models to improve motor performance, the suppression of this oscillation observed in different areas for subjects in the NO and HR levels of sleep-related fatigue could indicate the weakening in such ability during fatigue. The γ rhythm suppression could also suggest a weakening in the complex cognitive functions related to attention and memory (Jensen et al., 2007) expressed, for instance, in a difficult of maintaining visual shapes in short-term memory (Tallon-Baudry et al., 1998), reasonable for fatigued subjects (DP is higher than 2 in both levels).

The θ frequency is related to cognitive control. The increase of θ power is to coordinate activities of various brain regions to update the motor plan in response to somatosensory inputs. There is a suppression of this oscillation for subjects in the NO level and augmentation for the HR level. This could show the increase of the drowsy drivers' efforts to maintain the similar driving performance. This significant increase in the θ activity was also observed in drivers during the transitional phase from alertness to fatigue (Lal and Craig, 2002), in the frontal area was associated with mental fatigue (Wascher et al., 2014) and in the occipital-parietal areas was related to working-memory processing (Raghavachari et al., 2006).

We observed a suppression in the θ and α activities in the occipital area for subjects in the NO level of sleep-related fatigue. This finding suggests that the driver is more concentrated on the task than the ones in the HR level, for instance, processing some visual or auditory information from the realistic simulated vehicle, as observed in Lin et al. (2010). For subjects in the HR level, θ and α are activated in the occipital, motor and parietal areas (by the sources Frontal Midline and Parietal Midline). In this level of sleep-related fatigue representing a lack of sleep, the subjects tend more to mind-wandering under low perceptual demands (Lin et al., 2016). Similar findings were obtained during simulated driving in Huang et al. (2009).

The opposite trends in the change of α and β activities in the parietal area between subjects in those two sleep-related fatigue levels can be associated with different mechanisms for movement processing. In this context, subjects in the HR level could be more sensitive to movement selection demands where an increasing α and decreasing β were detected. Those findings are consistent with the actual and imagined movements reported in Brinkman et al. (2014).

The identification of distinct sleep-related fatigue levels was crucial for discriminating the effective connectivity patterns observed in the task-positive network of drivers. Their importance is based on the hypothesis that the sleep loss may affect brain functions locally, in a bottom-up regulation of temporal changes in neurobehavioral performance (Van Dongen et al., 2011), suggesting a dependence on cumulative increase in activation of the neuronal groups. This summative activation requiring to gather cognitive resources can explain the neural network changes observed in different frequencies during the sustained-attention driving task. Our results from DP, normalized CCM and spectral values support this bottom-up theory where performance is readjusted by the circadian rhythm and time-on-task effects.

## 5. Conclusion

The combination of EEG, behavioral and physiological information (expressed respectively in the CCM, DP and ES measures) as well the information about the task and socio-environmental context in which the driving experiments were performed, can highlight the real-world fatigue phenomenon. The spectral changes observed in the alertness oscillations can be explained by effective connectivity measures. CCM analysis over specific brain areas brain areas can predict different patterns of augmentation and suppression in the neural rhythms. CCM results can improve the development of real time devices for monitoring driver vigilance.

## Author contributions

AF and T-PJ contributed conception and design of the study. C-TL and J-TK organized the database. AF and SK performed the statistical analysis. AF wrote the first draft of the manuscript. SK wrote sections of the manuscript. All authors contributed to manuscript revision, read and approved the submitted version.

### Conflict of interest statement

The authors declare that the research was conducted in the absence of any commercial or financial relationships that could be construed as a potential conflict of interest. The reviewer YA and handling Editor declared their shared affiliation.
