# Supplementary material for: Brain Network Changes in Fatigued Drivers: A Longitudinal Study in a Real-World Environment Based on the Effective Connectivity Analysis and Actigraphy Data
Source: Front Hum Neurosci. 2018 Nov 12;12:418. doi: 10.3389/fnhum.2018.00418 (PMC6240698; doi:10.3389/fnhum.2018.00418)
Supplement: Supplementary file 1 [file Data_Sheet_1.PDF]

Supplementary Material: Brain network changes in fatigued drivers: a longitudinal study in a real-world environment based on the effective connectivity analysis and actigraphy data

1 ARTIFACT REJECTION

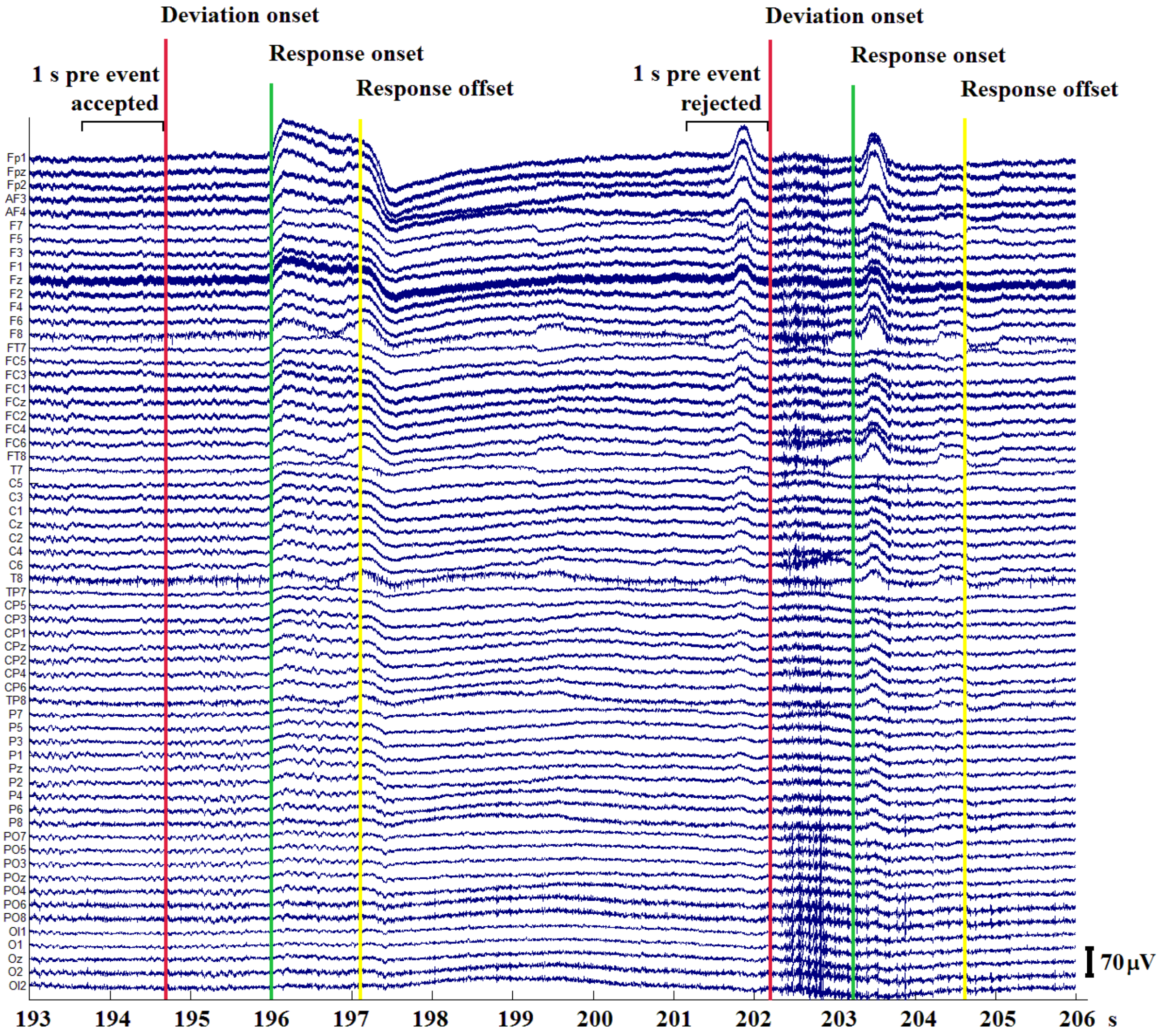

Figure S1: Examples of accepted and rejected epochs in the EEG data from the Lane Keeping Task.

2 CCM EFFICIENCY TEST

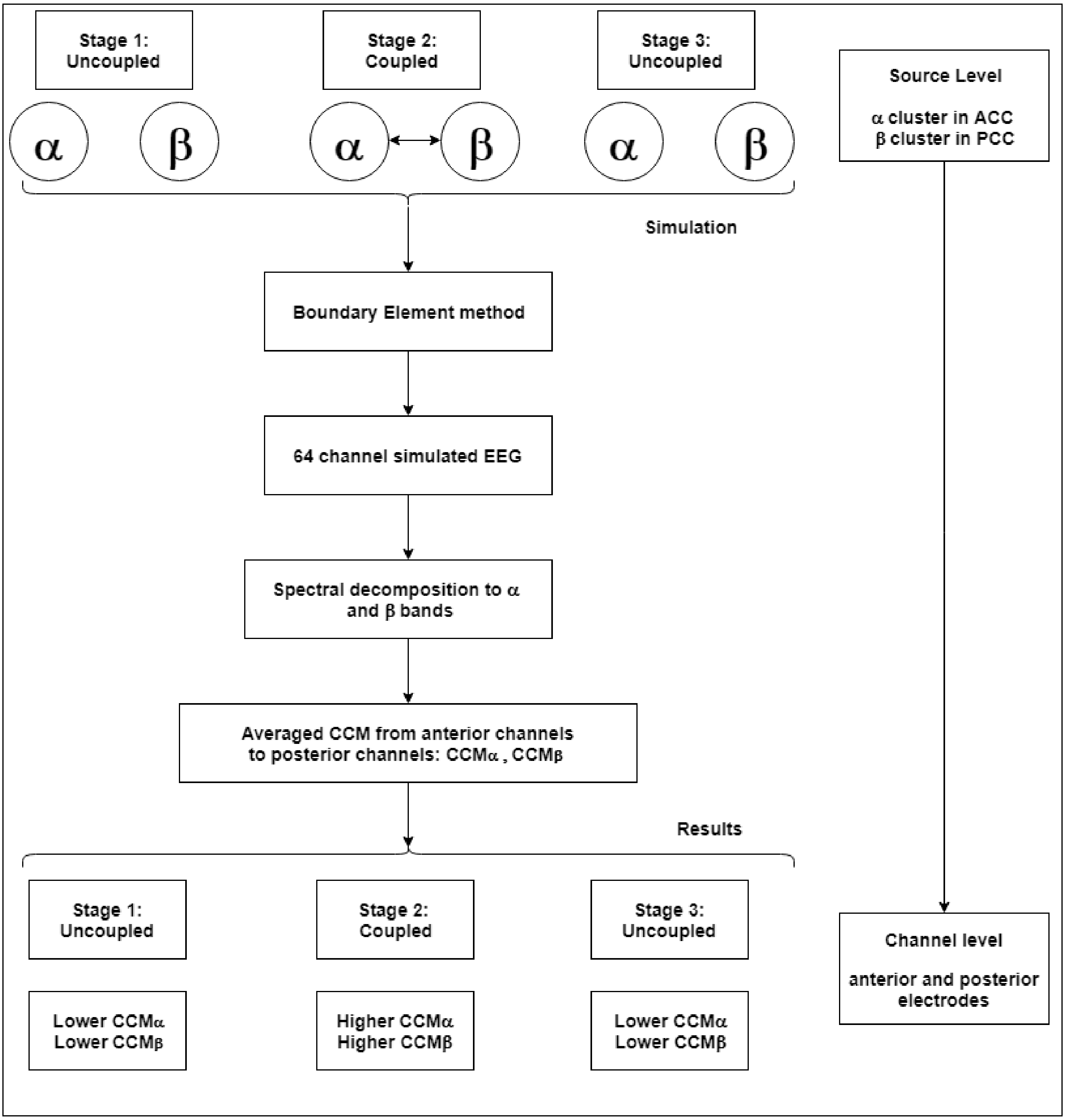

Figure S2: Flowchart of the brain connectivity model simulation study.

3 MAIN SOURCE CODES

The full source codes are available upon request to academic users. Contact: andre.fonseca@ufabc.edu.br.

3.1 SIFT - Prototype VAR structure

```
% Atlas and sampling rate
HeadModelPath = 'Colin27_Biosemi_1010_standard.mat';
SamplingRate = 200;
dt=5;

% Stages
S1_width = 20*SamplingRate;
S2_width = 20*SamplingRate;
S3_width = 20*SamplingRate;
Nl = S1_width+S2_width+S3_width;
Nr = 1;
ndisc = 0;
ModelOrder = 5;
S1_center = S1_width/2;
S2_center = S1_width+S2_width/2;
S3_center = S1_width+S2_width+S3_width/2;

% Equations
expr = { ...
sprintf('x1(t) = %s +0.01*x1(t-1) - 0.01*x2(t-2) - 0.01*x3(t-3) - 0.01*x4(t-4) + e1(t)', sim_dampedOscillator(8,dt,SamplingRate,1)) ...
sprintf('x2(t) = %s +0.01*x2(t-1) - 0.01*x1(t-2) - 0.01*x3(t-3) - 0.01*x4(t-4) + e2(t)', sim_dampedOscillator(10,dt+1,SamplingRate,2)) ...
sprintf('x3(t) = %s +1.5*x3(t-1) - 0.01*x1(t-2) + 0.01*x2(t-3) - 0.01*x4(t-4) +normpdfg(t,%f,%f,%f,40)*x5(t-5) + e3(t)', sim_dampedOscillator(10,dt-1,SamplingRate,3),S2_width/2,8,S2_center) ...
sprintf('x4(t) = %s +0.01*x4(t-1) - 0.01*x1(t-2) - 0.01*x2(t-3) - 0.01*x3(t-4) + e4(t)', sim_dampedOscillator(12,dt+2,SamplingRate,4)) ...
sprintf('x5(t) = %s -0.01*x5(t-1) - 0.01*x6(t-2) - 0.01*x7(t-3) - 0.01*x8(t-4) + e5(t)', sim_dampedOscillator(20,dt-2,SamplingRate,5)) ...
sprintf('x6(t) = %s -1.5*x6(t-1) + 0.01*x5(t-2) - 0.01*x7(t-3) + 0.01*x8(t-4) +normpdfg(t,%f,%f,%f,40)*x4(t-5) + e6(t)', sim_dampedOscillator(22,dt+3,SamplingRate,6),S2_width/2,8,S2_center) ...
```

sprintf('x7(t) = %s -0.01*x7(t-1) - 0.01*x5(t-2) - 0.01*x6(t-3) - 0.01*x8(t-4) + e7(t)', sim_dampedOscillator(25,dt-3,SamplingRate,7)) ...

sprintf('x8(t) = %s -0.01*x8(t-1) - 0.01*x5(t-2) - 0.01*x6(t-3) - 0.01*x7(t-4) + e8(t)', sim_dampedOscillator(30,dt-4,SamplingRate,8)) ...

};

proto_var_struc = sim_genVARModelFromEq(expr,ModelOrder);

3.2 SIFT - VAR process simulation and data generation

[A] = sim_genTVARcoeffs(proto_var_struc,ModelOrder,Nl,'NumSamplesToDiscard',ndisc,'Verbose',true);

sigma = 1;

M = size(A{1},1);

C = sigma*eye(M);

data = tvarsim(zeros(1,M),A,C,[Nl Nr],ndisc,2,1,'gengauss ');

3.3 SIFT - Forward Model

hmObj = hlp_validateHeadModelObject(HeadModelPath);

roiNames = hmObj.atlas.label;

roiIndex = [3 4 5 6 53 54 51 52];

roiNames = roiNames(roiIndex);

% 3 - G_and_S_cingul-Ant L ;

% 4 - G_and_S_cingul-Ant R ;

% 5 - G_and_S_cingul-Mid-Ant L ;

% 6 - G_and_S_cingul-Mid-Ant R ;

%51 - G_pariet_inf-Supramar L ;

%52 - G_pariet_inf-Supramar R ;

%53 - G_parietal_sup L ;

%54 - G_parietal_sup R.

[EEG,~, fwdModel] = sim_eegdata('srcdyn',{'arg_selection' 'Precomputed' 'srcdata' data}, ...

'fwdproj', {'hmObj' hmObj 'sourceAtlasLabels' {} 'LFM' [] 'channels' {} ...

'sourceShape' {'arg_selection' 'dipole' 'roiAtlasLabels' {} 'roiOrdered ' roiNames 'nearestNeighbor' true}, ...

'addNoise', {'SignalToNoise' 4}, 'verb' 2},'makedipfit',true,'vismodel',false,'verb',2);

3.4 Reconstruction

```
function [xr] = takens(x, m, tau)
% INPUT
% x : scalar time series
% m : embedding dimension
% tau : time lag
% OUTPUT
% xr : reconstructed phase space

for i = 1 : length(x) - (m - 1) * tau
    xr(i, 1 : m) = x(i : tau : i + (m - 1) * tau);
end
end
```

3.5 CCM

```
function [causa_corr] = causal (x,y,m,tau)
% INPUT
% x : scalar time series (expected to cause y)
% y : scalar time series (expected to be caused by x)
% m : embedding dimension for reconstruction
% tau: time lag for reconstruction
% OUTPUT
% causa_corr: correlation between y and the y_hat (predicted values using x)

% same size
minsize=min(length(x),length(y));
if (length(x)~=minsize) || (length(y)~=minsize)
    x=x(1:minsize);
    y=y(1:minsize);
end

% Reconstruction and size
xvec=takens(x,m,tau);
```

```
N=minsize-(m-1)*tau;

parfor pos=1:N

    % euclidian distances to xvec(pos,:)
    dist = sqrt(sum((repmat(xvec(pos,:),N,1)-xvec).^2,2));

    % no self-match
    index=find(dist~=0);

    u=exp(-dist(index)/min(dist(index)));
    w=u./sum(u);
    y_hat(pos)=w'*y(index)';

end

causa_corr=corrcoef(y(end-N:end-1),y_hat);
causa_corr=causa_corr(1,2);

end
```

4 EVALUATIONS AND STATISTICS

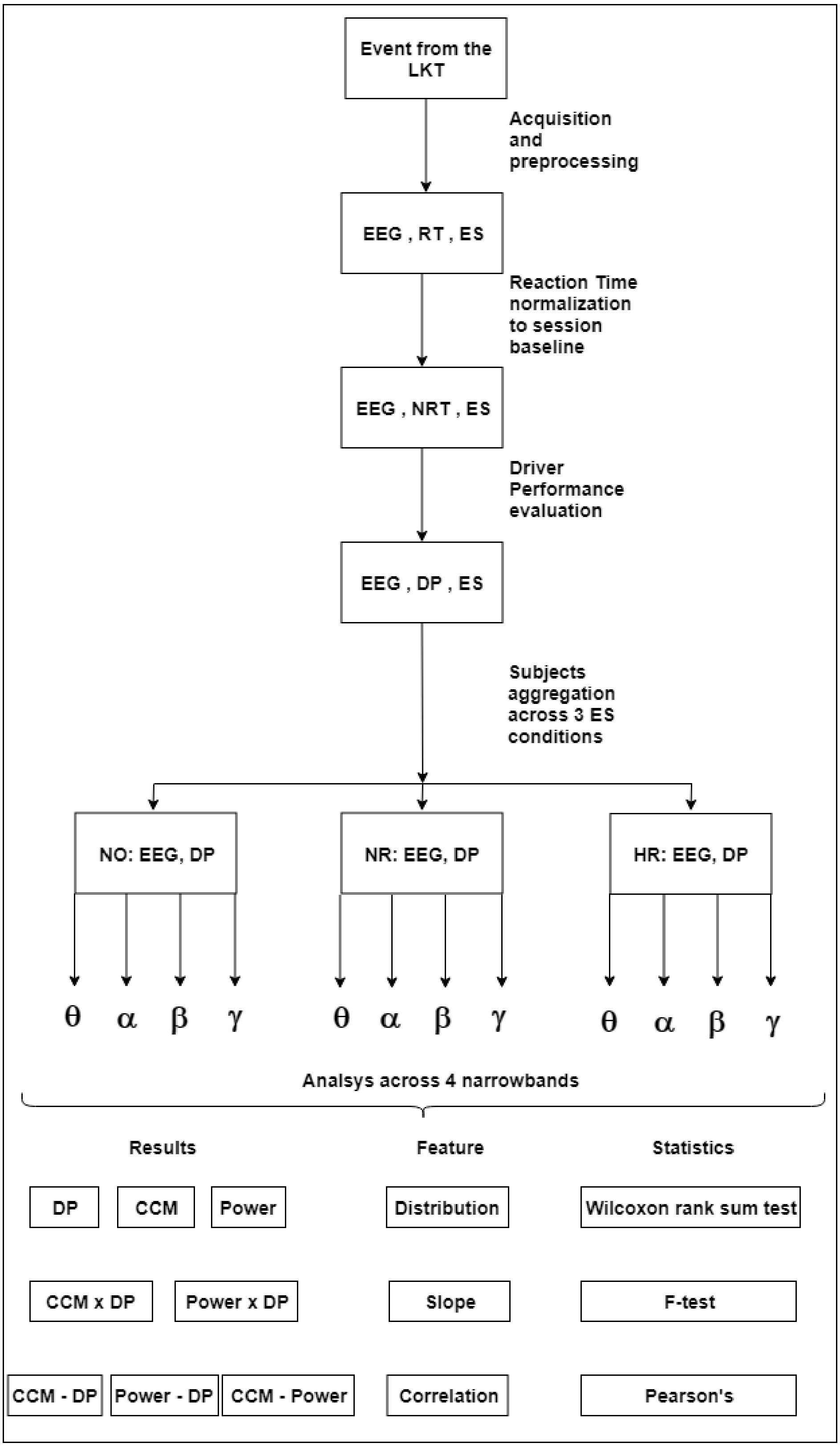

Figure S3: Flowchart of the overall process.
